# Superior predictive value of estimated pulse wave velocity for all-cause and cardiovascular disease mortality risk in U.S. general adults

**DOI:** 10.1186/s12889-024-18071-2

**Published:** 2024-02-24

**Authors:** Wenke Cheng, Fanliang Kong, Huachun Pan, Sisi Luan, Shumin Yang, Siwei Chen

**Affiliations:** 1 Medical Faculty, University of Leipzig, Leipzig, Germany; 2 University Medical Center of Göttingen, Georg-August University, Göttingen, Germany; 3 College of Veterinary Medicine, Huazhong Agricultural University, Wuhan, China; 4 Department of Endocrinology, Beijing Tongren Hospital, Capital Medical University, Beijing, China; 5 State Key Laboratory of Agriculture Microbiology, College of Veterinary Medicine, Huazhong Agricultural University, Wuhan, China; 6 Department of Cardiovascular Medicine, Nanchang People’s Hospital (The Third Hospital of Nanchang), No.1268, Jiuzhou Street, Chaoyang New District, Jiangxi, Nanchang China

**Keywords:** Estimated pulse wave velocity, National Health and Nutrition Examination Survey, All-cause mortality, Cardiovascular disease, Mortality

## Abstract

**Background:**

Estimated pulse wave velocity (ePWV) has been proposed as a potential approach to estimate carotid-femoral pulse wave velocity. However, the potential of ePWV in predicting all-cause mortality (ACM) and cardiovascular disease mortality (CVM) in the general population is unclear.

**Methods:**

We conducted a prospective cohort study using the data of 33,930 adults (age ≥ 20 years) from the National Health and Nutrition Examination Survey (NHANES) from 1999 to 2014 until the end of December 2019. The study outcomes included ACM and CVM. Survey-weighted Cox proportional hazards models were used to assess hazard ratios (HRs) and 95% confidence intervals (CIs) to determine the association between ePWV and ACM and CVM. To further investigate whether ePWV was superior to traditional risk factors in predicting ACM and CVM, comparisons between ePWV and the Framingham Risk Score (FRS) and Pooled Cohort Equations (PCE) models were performed. Integrated Discriminant Improvement (IDI) and Net Reclassification Improvement (NRI) were employed to analyze differences in predictive ability between models.

**Results:**

The weighted mean age of the 33,930 adults included was 45.2 years, and 50.28% of all participants were men. In the fully adjusted Cox regression model, each 1 m/s increase in ePWV was associated with 50% and 49% increases in the risk of ACM (HR 1.50; 95% CI, 1.45–1.54) and CVM (HR 1.49; 95% CI, 1.41–1.57), respectively. After adjusting for FRS, each 1 m/s increase in ePWV was still associated with 29% (HR 1.29; 95% CI, 1.24–1.34) and 34% (HR 1.34; 95% CI, 1.23–1.45) increases in the risk of ACM and CVM, respectively. The area under the curve (AUC) predicted by ePWV for 10-year ACM and CVM were 0.822 and 0.835, respectively. Compared with the FRS model, the ePWV model improved the predictive value of ACM and CVM by 5.1% and 3.8%, respectively, with no further improvement in event classification. In comparison with the PCE model, the ePWV model’s ability to predict 10-year ACM and CVM was improved by 5.1% and 3.5%, and event classification improvement was improved by 34.5% and 37.4%.

**Conclusions:**

In the U.S. adults, ePWV is an independent risk factor for ACM and CVM and is independent of traditional risk factors. In the general population aged 20 to 85 years, ePWV has a robust predictive value for the risk of ACM and CVM, superior to the FRS and PCE models. The predictive power of ePWV likely originates from the traditional risk factors incorporated into its calculation, rather than from an indirect association with measured pulse wave velocity.

**Supplementary Information:**

The online version contains supplementary material available at 10.1186/s12889-024-18071-2.

## Introduction

Arterial stiffness is a hallmark of the aging process and an essential manifestation of vascular aging [1]. The formation of arterial stiffness is characterized by collagen and calcium deposition or hemodynamics-induced elastin disruption, which alters the thickness and function of the arterial wall and elastin, affecting vascular tone and compliance, and is a vital determinant of multi-organ damage [2, 3]. Several studies have indicated that arterial stiffness contributes to the onset of various diseases, such as cardiovascular disease (CVD), cerebrovascular disease, and diabetes mellitus (DM) [4–7]. Therefore, recently, arterial stiffness measurement has gained increasing recognition, not only as a means of assessing disease risk but also as a way of facilitating complementary interventions to reverse this trend.

Arterial stiffness generally increases progressively from the heart to the periphery, indicating that aortic stiffness may occur first [8]. Carotid-femoral pulse wave velocity (cfPWV) is currently recommended as the gold standard for the evaluation of central arterial stiffness [9]. However, cfPWV measurement requires specific equipment, specialized technical skills, and special procedures limiting its clinical application [10]. Therefore, it is urgent to discover a simple and reproducible method to promote its applicability in clinical practice to estimate the degree of aortic stiffness. Recently, Vlachopoulos et al*.* introduced a potential alternative to cfPWV, namely, estimated pulse wave velocity (ePWV), which has been found to predict the risk of cardiovascular outcomes independent of traditional CVD risk factors [11]. Additionally, Heffernan et al*.* demonstrated that ePWV independently predicted the risk of all-cause mortality (ACM) and cardiovascular mortality (CVM) in the general population without CVD [12]. In this study, a larger national sample was used to investigate whether ePWV predicted ACM and CVM in the general population.

## Methods

### Study design and population

The study was a prospective cohort study that utilized data from the National Health and Nutrition Examination Survey (NHANES) between 1999 and 2014, with follow-up until December 2019. NHANES, a publicly accessible database in the United States, is a complex, stratified, multistage probability survey conducted by the National Center for Health Statistics including interviews, physical examinations at home or mobile examination centers (MEC), and laboratory tests, and is carried out every two years. The survey targets the civilian, noninstitutionalized population in the US, and is nationally representative. Detailed sampling and data collection procedures have been previously published [13]. The NHANES was conducted by the National Center for Health Statistics of the US Centers for Disease Control and Prevention (CDC) and approved by the Institutional Review Board of the National Center for Health Statistics, with written informed consent obtained from all participants.

The study included a total of 82,091 US individuals, among which 43,793 were adults aged over 20 years who participated in eight NHANES survey cycles between 1999 and 2014. Of them, 9156 adults were excluded due to missing weight data, follow-up or ePWV data, pregnancy, or cancer. To lower the potential for reverse causation bias, 707 individuals who died within two years of follow-up were also excluded. Finally, 33,930 adults were involved in the analysis (Figure S1).

### Calculation of ePWV

The ePWV was calculated using a formula first described by Greve et al*.* and derived by the Arterial Stiffness' Collaboration [14, 15]. The formula is as follows:

$$\mathrm{ePWV}\;=\;9.587-\left(0.402\;\times\;\mathrm{age}\right)\;+\;\left[4.560\;\times\;10^{-3}\;\times\;\left(\mathrm{age}^2\right)\right]-\left[2.621\;\times\;10^{-5}\;\times\;\left(\mathrm{age}^2\right)\;\times\;\mathrm{mean}\;\mathrm{blood}\;\mathrm{pressure}\;\left(\mathrm{MBP}\right)\right]\;+\;3.176\;\times\;10^{-3}\;\times\;\mathrm{age}\;\times\;\mathrm{MBP}-1.832\;\times\;10^{-2}\;\times\;\mathrm{MBP}$$

where age is in years and mean blood pressure (MBP) is calculated by diastolic blood pressure (DBP) + 0.4 × [systolic blood pressure (SBP)—DBP]. To record blood pressure, participants were seated still for five min, and a trained examiner employed a standard sphygmomanometer to measure blood pressure. Almost all the participants chose the right arm (> 99%) and only a very small percentage chose the left arm (< 1%). The average of at least three readings was considered. The blood pressure measurement technique was in accordance with the latest American Heart Association recommendations for human blood pressure measurement. Detailed information about the quality assurance and quality control process is presented in the physician section of the MEC Operations Manual [16].

### Definition of outcomes

The study assessed two main outcomes: ACM and CVM. The outcomes were determined by linking the study data to the National Mortality Index until December 2019. ACM represented deaths resulting from all causes, while CDM was identified using ICD-10 codes I00-I09, I11, I13, I20-I51.

### Other variables of interest

In addition to the variables needed for computing ePWV, several other variables were considered in this study. Standardized questionnaires were conducted to collect information on age, gender, race, education level, family income, smoking and drinking status, medical history, medication use, Framingham Risk Score (FRS), and Pooled Cohort Equations (PCE). Participants reported their medical history based on previous medical records from a healthcare professional or physician. CVD was defined as a group of diseases, including coronary heart disease, congestive heart failure, heart attack, stroke, and angina pectoris. Biochemical parameters were measured based on a rigorous procedure, with details provided in the NHANES Procedures Manual for Laboratory/Medical Technologists [16]. At the MEC, all participants underwent blood pressure, weight, and height measurements. The following variables were further classified to facilitate data integration:

i) Race: non-Hispanic white people, non-Hispanic black people, Mexican Americans, or other races.
ii) Educational level: Less than 9th grade, 9-11th grade/high school or equivalent, college graduate or above
iii) Smoking status: Never (< 100 cigarettes/lifetime), former smoker (> 100 cigarettes/lifetime and absolutely no smoking now), or current smoker (> 100 cigarettes/lifetime and currently smoking some days or every day) [17].
iv) Drinking status: Never (< 12 drinks/lifetime), former drinker (≥ 12 drinks/lifetime but not in the past year), current light/moderate drinker (≤ 1 drink/day for women and ≤ 2 drinks/day for men in the past year), or current heavy drinker (> 1 drink/day for women and > 2 drinks/day for men in the past year) [18].
v) The FRS is a comprehensive assessment analyzing multiple CVD risk factors, such as age, gender, smoking status, blood pressure, total cholesterol levels, and high-density lipoprotein levels. It estimates the probability of an individual experiencing CVD events within the next 10 years. Through incorporating these risk factors into an overall score, individuals can be classified into three CVD risk categories based on their likelihood of encountering a CVD event within 10 years: low risk (< 10%), moderate risk (10–20%), and high risk (≥ 20%) [19].
vi) The pooled cohort equations are a risk model developed in the multiracial United States by the American Heart Association (AHA) and the American College of Cardiology (ACC). Participants' 10-year CVD risk is calculated based on revised PCE, and scores are classified as low/borderline (< 7.5%), moderate (7.5–20%), and high (≥ 20%) following the 2018 ACC/AHA cholesterol guidelines [20, 21].

### Statistical analysis

Appropriate weights (MEC weights) were used to account for oversampling, non-response, and non-coverage, and to provide nationally representative estimates. A detailed description of the weighting methods is available on the NHANES website (https://wwwn.cdc.gov/nchs/nhanes/tutorials/Module3.aspx).

Continuous variables were shown as means (standard error, SE) for baseline demographic characteristics, and categorical variables were presented as unweighted counts (weighted %). ePWV, as a continuous variable, was divided into quartiles, and Schoenfeld Residuals were applied to test the proportional hazards assumption. The cumulative hazard risk of the general population with different ePWV levels during the observation period was explored using Kaplan–Meier analysis with the log-rank test. Hazard ratios (HRs) and 95% confidence intervals (CIs) for the association between ePWV and ACM and CVM were calculated based on Cox proportional hazards models. Baseline variables were considered as candidate predictors for the multiple regression model.

To address the possibility of overfitting, the variance inflation factor (VIF) was used to quantify the extent of multicollinearity between variables. Variables with a VIF ≥ 10 were excluded [22]. Confounding covariates were classified and progressively added to the models. In addition, we also imputed missing variables and conducted pooled analyses of the Cox regression model, using a five-repeat predictive mean matching algorithm and the Markov chain Monte Carlo method, as a form of sensitivity analysis [23].

Subgroup analyses were performed stratified by the following clinical characteristics: sex (male, female), age (< 55, ≥ 55 years), body-mass index (< 30, ≥ 30 kg/m^2^), race (non-Hispanic white people, non-Hispanic black people, Mexican Americans, and other), asthma (no/yes), CVD (no/yes), arthritis (no/yes), CVD (no/yes), hypertension (no/yes), chronic bronchitis (no/yes), chronic kidney disease (CKD) (no/yes), drinking and smoking status; the P values for the interactions were obtained. Restricted cubic splines (RCS) were used to visually assess the dose–response relationship between ePWV and the risk of mortality. The P-value for non-linearity was obtained using the log-likelihood ratio test. As a complementary analysis, the relationship between MBP and risk of mortality was assessed. In addition, we performed a threshold effect analysis to examine how the risk of ACM and CVM changes with an increase in specific units of ePWV. If a non-linear association was observed, a two-piecewise linear regression model was conducted to determine the inflection point at which the relationship between ePWV and mortality significantly changed in the RCS [24].

To evaluate the predictive value of ePWV for 10-year ACM and CVM in the general population, the nearest neighbor estimation method was employed to plot time-dependent receiver operating characteristic (ROC) curves [25]. This approach enabled us to assess the ability of ePWV to discriminate between individuals who developed ACM or CVM within the 10-year time frame and those who did not. To further investigate whether ePWV outperformed traditional risk factors in predicting ACM and CVM, we compared ePWV with the FRS and PCE models from two cohorts. The first cohort was in the overall population aged between 20–85 years with or without a history of CVD. The second cohort was restricted to the population aged 30–74 years and 40–79 years without a history of CVD, based on the specific populations to which the FRS and PCE models were applied. Considering that blood pressure and age are key parameters in calculating ePWV, we further analyzed their predictive superiority for ACM and CVM in comparison to ePWV. Specifically, we independently compared the predictive effects of age, age squared, DBP, SBP, and MBP with those of ePWV. Additionally, we compared the predictive value of age in combination with its square and blood pressure (DBP/SBP), as well as age in combination with its square and MBP against ePWV. The Harrell's C-statistic (C-index) was used to measure the discriminatory ability of the models. Integrated discrimination improvement (IDI) was applied to assess the difference between the ePWV model and the FRS and PEC models in terms of the accuracy of mortality prediction [26]. Net reclassification improvement (NRI) was utilized to determine the improvement of the ePWV model for event classification relative to the FRS and PEC models [27]. The median improvement in risk score was calculated for all participants [27]. All analyses were performed using the statistical packages R (http://www.R-project.org, The R Foundation) and EmpowerStats (version 4.2.0, www.R-project.org, X&Y Solutions, Inc., Boston, MA). Two-sided *P*-values < 0.05 represented statistical significance.

## Results

From 1999 to 2014, a total of 33,930 general adults were enrolled in the NHANES. Their weighted mean age was 45.2 years, and 50.28% of the participants were men. The weighted overall demographic characteristics are presented in Table 1. During a median follow-up period of 133 months (interquartile range 94–184 months; 4,740,037 person-years), 5,138 all-cause and 1,386 CVD deaths were recorded.

**Table 1:** Survey-weighted baseline characteristics of the U.S. adults from NHANES 1999 to 2014 (*N* = 33,930, representing 19,472,771 individuals)

**ePWV**	7.89 (0.02)
**Demographic**	
Age (years)	45.2 (0.19)
Heart rate	72.5 (0.13)
Pulse pressure (mmHg)	50.06 (0.2)
Male	16,908 (50.28)
Race	
Non-Hispanic white	15,003(68.14)
Non-Hispanic black	7,263 (11.47)
Mexican American	6,465 (8.5)
Other races	5,199 (11.89)
Poverty income ratio	2.98 (0.03)
**Parameters**	
Body mass index (kg/m^2^)	28.47 (0.07)
Waist (cm)	97.31 (0.18)
Estimated glomerular filtration rate (mL/min/1.73 m^2^)	95.40 (0.27)
Total cholesterol (mmol/L)	5.11 (0.01)
High-density lipoprotein cholesterol (mmol/L)	1.36 (0.00)
**History of diseases**	
Cardiovascular diseases	3,231(7.33)
Chronic kidney disease	5,459(12.96)
Diabetes mellitus	5,235(10.90)
Chronic bronchitis	1,807(5.55)
Hypertension	13,803(35.15)
Arthritis	8,202(21.91)
**Medication**	
Antihypertensives	3,401 (8.55)
Glucose-lowering drugs	3,165 (6.45)
**Lifestyle**	
**Smoking**	
Never	18,346 (23.18)
Former	7913 (53.70)
Current	7643 (23.12)
** Drinking**	
Never	4,445 (11.51)
Former	5,768 (15.27)
Mild/Moderate	10,013 (34.27)
Heavy	11,200 (38.65)
**Cardiovascular risk models**	
**Framingham Risk Score (%)**	6.58 (0.07)
**Pooled Cohort Equations (%)**	3.8 (0.1)

Continuous variables are expressed as weighted mean (Standard error, SE)

Categorical variables are expressed as counts (weighted %)

Pulse pressure = systolic blood pressure—diastolic blood pressure

*NHANES* National Health and Nutrition Examination Survey, *ePWV* Estimated pulse wave velocity

### ePWV and all-cause mortality

**Fig. 1:**
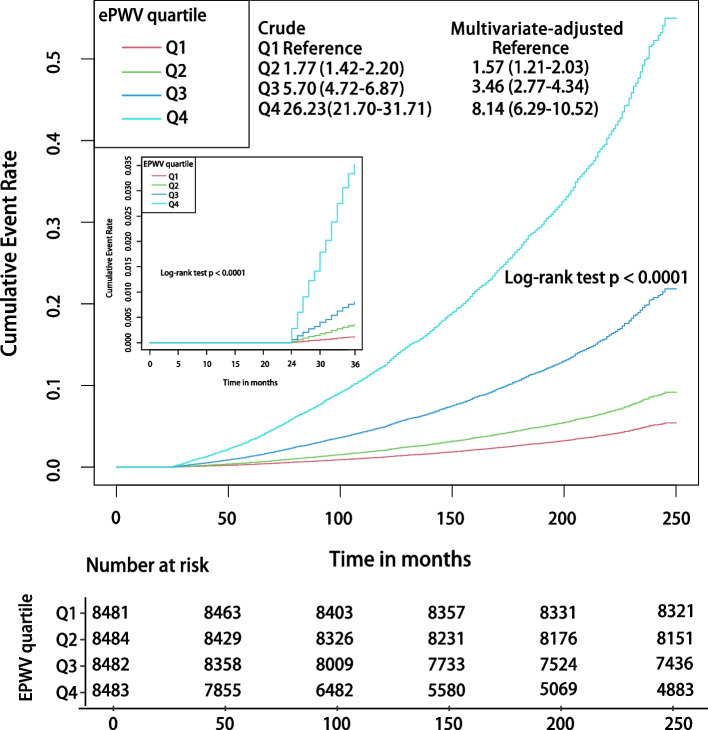
Kaplan–Meier survival curves, by ePWV quartile level, for all-cause mortality. Follow-up was initiated two years after enrollment, and landmark analyses were performed within one year of the start of follow-up. In the multivariate model the HRs have been fully adjusted for heart rate, pulse pressure, race, gender, poverty income ratio, body mass index, waist, estimated glomerular filtration rate, total cholesterol, high-density lipoprotein cholesterol, cardiovascular diseases, chronic kidney disease, diabetes mellitus, chronic bronchitis, hypertension, Arthritis, antihypertensives, glucose-lowering drugs, smoking, and drinking

Kaplan–Meier curves showed that there was a graded positive association between quartile increases in ePWV and the risk of ACM, both within the first year of follow-up and throughout the study period (all *P* < 0.001 by log-rank test). The graded positive association between increasing quartiles of ePWV and risk of ACM was in the crude model (HR for ePWV in Q 4 and Q 1: 26.33; 95% CI: 21.70–31.71) and the multivariable-adjusted model (HR for ePWV in Q 4 and Q 1: 8.14; 95% CI: 6.29–10.52) (Fig. 1). Very similar results were obtained in the cohorts excluded for only one year (all *P* < 0.001 by log-rank test). The risk of ACM was increased significantly as the level of ePWV quartiles was increased stepwise (Figure S2).

In the unadjusted cox regression model, every 1 m/s increase in ePWV was associated with a 67% increase in the risk of ACM (HR 1.67; 95% CI, 1.65–1.70; *P* < 0.001) (Table 2). Likewise, this hazard risk remained after adjusting for heart and pulse pressure (HR 1.66; 95% CI, 1.62–1.70; *P *< 0.001). In the model fully adjusted for confounders, every 1 m/s increase in ePWV was associated with a 50% increase in the risk of ACM (HR 1.50; 95% CI, 1.45–1.54; *P* < 0.001), which was comparable to the obtained results after multiple imputations (HR 1.48; 95% CI, 1.44–1.53; *P* < 0.001) (Table S1). After adjusting for FRS, the risk of ACM still increased by 29% (HR 1.29; 95% CI, 1.24–1.34; *P* < 0.001) for every 1 m/s increase in ePWV. After additional adjustment for PCE, the risk of ACM increased by 30% (HR 1.30; 95% CI, 1.24–1.35; *P* < 0.001) for each 1 m/s increase in ePWV. In sensitivity analyses, whether pulse pressure was replaced with systolic and diastolic blood pressure or age was also considered in multivariate models, and an increase in ePWV was significantly and positively associated with the risk of ACM (Table S2).

**Table 2:** Survey-weighted cox proportional hazard results examining the association of ePWV on all-cause and CVD mortality in the general U.S. adults from NHANES 1999 to 2014

	**Death**	**Unadjusted Model**	**Adjusted Model 1**	**Adjusted Model 2**	**Adjusted Model 3**	**Adjusted Model 4**	**Adjusted Model 5**	**Adjusted Model 6**
**ePWV, 1 m/s increase**		**HR (95%CI)**	**HR (95%CI)**	**HR (95%CI)**	**HR (95%CI)**	**HR (95%CI)**	**HR (95%CI)**	**HR (95%CI)**
All-cause Mortality	5,138	1.67 (1.65–1.70)^a^	1.66 (1.62–1.70)^a^	1.64 (1.61–1.68)^a^	1.48 (1.45–1.52)^a^	1.45 (1.41–1.50)^a^	1.46 (1.42–1.50)^a^	1.50 (1.45–1.54)^a^
CVD Mortality	1,386	1.78 (1.74–1.82)^a^	1.72 (1.65–1.79)^a^	1.71 (1.65–1.78)^a^	1.51 (1.44–1.58)^a^	1.45 (1.38–1.53)^a^	1.46 (1.39–1.54)^a^	1.49 (1.41–1.57)^a^
**ePWV, 1 m/s increase**		**Adjusted Model 7**	**Adjusted Model 8**					
		**HR (95%CI)**	**HR (95%CI)**					
All-cause Mortality	5,138	1.29 (1.24–1.34)^a^	1.30 (1.24–1.35)^a^					
CVD Mortality	1,386	1.34 (1.23–1.45)^a^	1.35 (1.24–1.47)^a^					

Respiratory diseases indicated all deaths from chronic lower respiratory diseases. Renal disease indicated all deaths from nephritis, nephrotic syndrome, and nephrosis

Model 1 adjust heart rate(continuous), pulse pressure (continuous)

Model 2 adjust Model 1 plus other demographic variables including gender (male, gender), race (non-Hispanic white, non-Hispanic black, Mexican American, and other races) and poverty income ratio (continuous)

Model 3 adjusted Model 2 plus other parameters including body mass index (continuous), waist (continuous), estimated glomerular filtration rate (continuous), total cholesterol (continuous), and high-density lipoprotein cholesterol (continuous)

Model 4 adjusted Model 3 plus history of diseases including cardiovascular diseases (yes/no), chronic kidney disease (yes/no), diabetes mellitus (yes/no), chronic bronchitis (yes/no), hypertension (yes/no), and Arthritis (yes/no)

Model 5 adjusted Model 4 plus medication including antihypertensives (yes/no) and glucose-lowering drugs (yes/no)

Model 6 adjusted Model 5 plus lifestyle variables including smoking (never, former, and current), and drinking (never, former, mild/moderate, and heavy)

Model 7 adjusted Model 6 plus Framingham Risk Score

Model 8 adjusted Model 7 plus Pooled Cohort Equations

^a^indicates *p*-value < 0.001

**Fig. 2:**
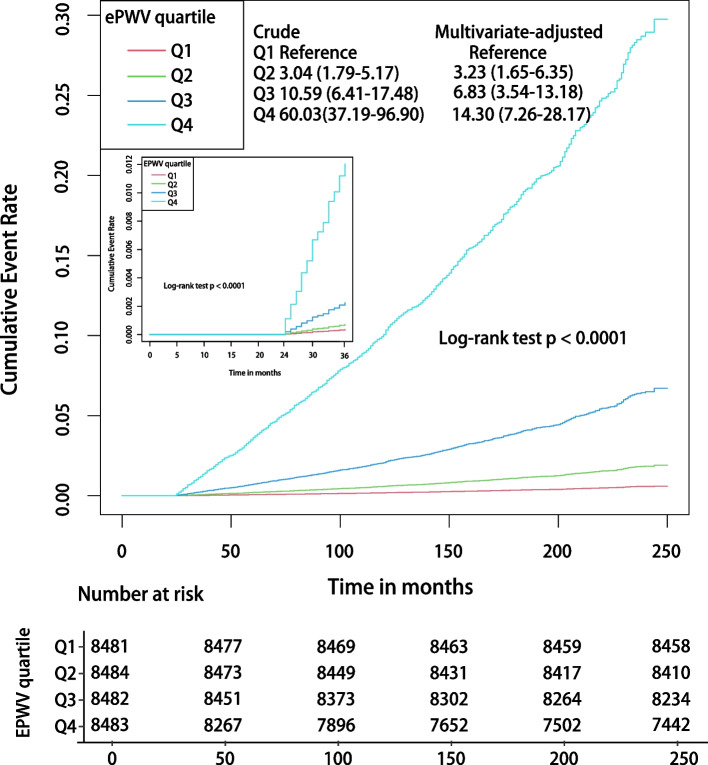
**A** Subgroup analyses of the associations (hazard ratios, 95% CIs) between ePWV values and the risk of all-cause mortality. HRs indicate the increased risk of all-cause mortality for each 1 m/s increase in ePWV. **B** Subgroup analyses of the associations (hazard ratios, 95% CIs) between ePWV values and the risk of CVD mortality. HRs have been fully adjusted for heart rate, pulse pressure, race, gender, poverty income ratio, body mass index, waist, estimated glomerular filtration rate, total cholesterol, high-density lipoprotein cholesterol, cardiovascular diseases, chronic kidney disease, diabetes mellitus, chronic bronchitis, hypertension, Arthritis, antihypertensives, glucose-lowering drugs, smoking, and drinking

In the subgroup analysis, the positive association between ePWV and the risk of ACM was consistent across all strata. Every 1 m/s increase in ePWV was in consistence with a 29%–59% increase in the risk of ACM (Fig. 2).

### ePWV and CVD mortality

Figure 2 shows a gradient positive association between increasing quartiles of ePWV and the risk of CVM, both within the first year of follow-up and throughout the observation period (all *P* < 0.001 by log-rank test). There existed a graded positive association in both the crude model (HR for ePWV in Q 4 and Q 1: 60.03; 95% CI: 37.19–96.90) and the multivariable-adjusted model (HR for ePWV in Q 4 and Q 1: 14.3; 95% CI: 7.26–28.17).

In the unadjusted cox regression model, every 1 m/s increase in ePWV was associated with a 78% increase in the risk of CVM (HR 1.78; 95% CI, 1.64–1.82; *P* < 0.001) (Table 2). In the model fully adjusted for confounders, every 1 m/s increase in ePWV was associated with a 49% increase in the risk of CVM (HR 1.49; 95% CI, 1.41–1.57; *P* < 0.001), which was comparable to the obtained results after multiple imputations (HR 1.48; 95% CI, 1.40–1.53; *P* < 0.001) (Table S1). After adjusting for FRS, with every 1 m/s increase in ePWV, the risk of CVD was still increased by 34% (HR 1.34; 95% CI, 1.23–1.45; *P* < 0.001). After additional adjustment for PCE, the risk of CVM was increased by 35% (HR 1.35; 95% CI, 1.24–1.47; *P* < 0.001) for each 1 m/s increase in ePWV. When sensitivity analyses were performed, an increase in ePWV was independently correlated with a higher CVM risk, either by replacing pulse pressure with systolic and diastolic blood pressure or by additionally considering age in multivariate models (Table S2).

**Fig. 3:**
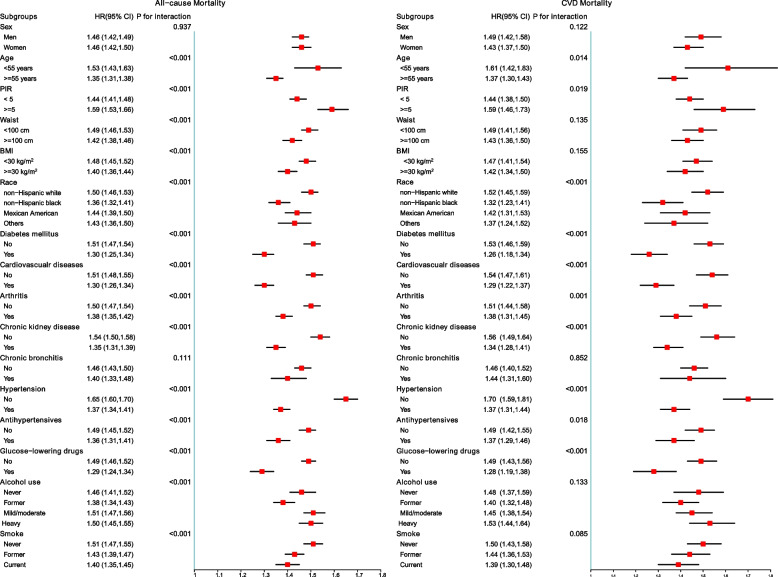
Kaplan–Meier survival curves, by ePWV quartile level, for CVD mortality. Follow-up was initiated two years after enrollment, and landmark analyses were performed within one year of the start of follow-up. In the multivariate model the HRs have been fully adjusted for heart rate, pulse pressure, race, gender, poverty income ratio, body mass index, waist, estimated glomerular filtration rate, total cholesterol, high-density lipoprotein cholesterol, cardiovascular diseases, chronic kidney disease, diabetes mellitus, chronic bronchitis, hypertension, Arthritis, antihypertensives, glucose-lowering drugs, smoking, and drinking

Subgroup analysis further strengthened the positive association between ePWV and the risk of CVM, which was consistent across all strata, with a 28% to 70% increase in the risk of CVM for each 1 m/s increase in ePWV (Fig. 3).

### Dose-dependent relationship

**Fig. 4:**
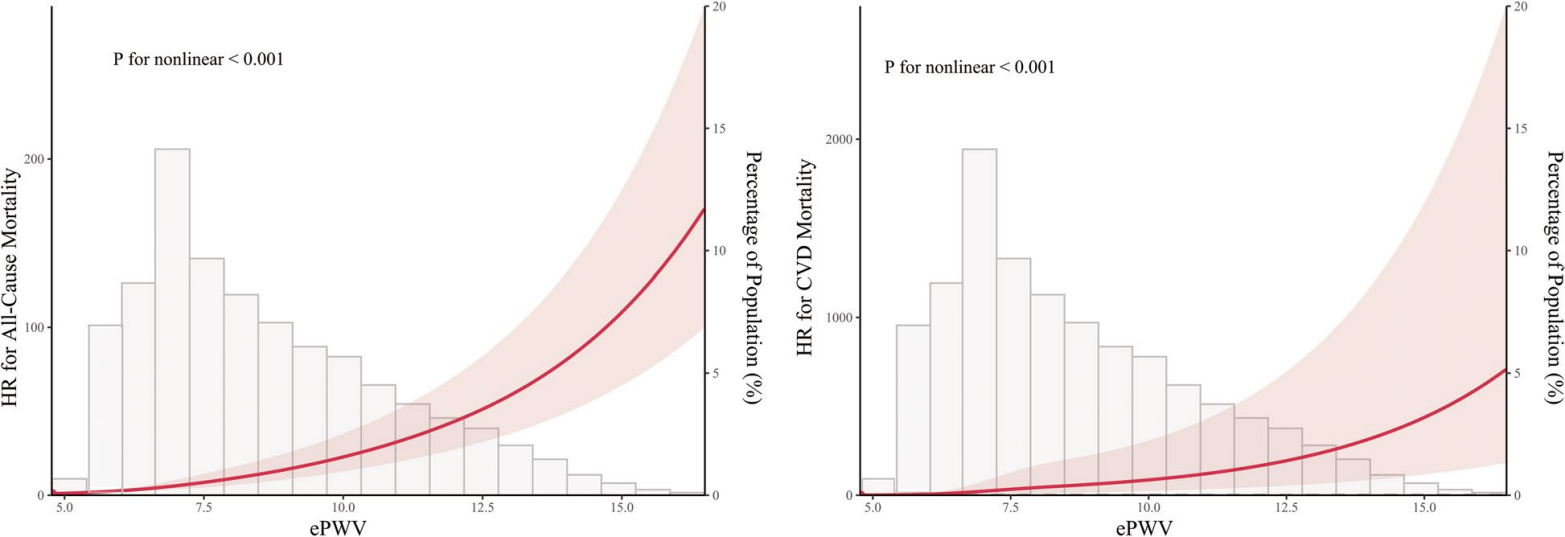
**A** Dose–response relationship between ePWV with the risk of all-cause mortality. **B** Dose–response relationship between ePWV with the risk of CVD mortality. HRs have been fully adjusted for heart rate, pulse pressure, race, gender, poverty income ratio, body mass index, waist, estimated glomerular filtration rate, total cholesterol, high-density lipoprotein cholesterol, cardiovascular diseases, chronic kidney disease, diabetes mellitus, chronic bronchitis, hypertension, Arthritis, antihypertensives, glucose-lowering drugs, smoking, and drinking

As shown in Fig. 4, the nonlinear correlation between ePWV levels and the risk of ACM was observed (P for nonlinear < 0.001), with an inflection point of 8.76 m/s. The threshold effect analysis demonstrated that the risk of ACM was increased by 89% (HR 1.89; 95% CI 1.77–2.02; *P *< 0.001) for every 1 m/s increase in ePWV when ePWV was < 8.76 m/s. However, when ePWV was ≥ 8.76 m/s, ACM was increased by 38% with every 1 m/s increase in ePWV (HR 1.38; 95% CI 1.34–1.41; *P* < 0.001) (Table S3). As shown in Fig. 4, ePWV levels were shown to be nonlinearly associated with CVM (P for nonlinear < 0.001). The threshold effect analysis revealed that the risk of CVM changed significantly when ePWV was < 7.57 m/s, with the risk of CVM increasing by 2.07-fold (HR 3.07; 95% CI 2.22–4.24; *P* < 0.001). However, when ePWV was ≥ 7.57 m/s, every 1 m/s increase in ePWV was related to a 40% increase in ACM (HR 1.40; 95% CI 1.34–1.46; *P* < 0.001) (Table S3).

In the meanwhile, we analyzed the relationship between MBP and ACM and CVM, finding that there was a U-shaped relationship between MBP and ACM with the inflection points at 82 and 100 mmHg, respectively (Figure S3). Table S4 shows the detailed stage-specific risk assessment.

### Predictive value of ePWV for 10-year ACM and CVM

**Fig. 5:**
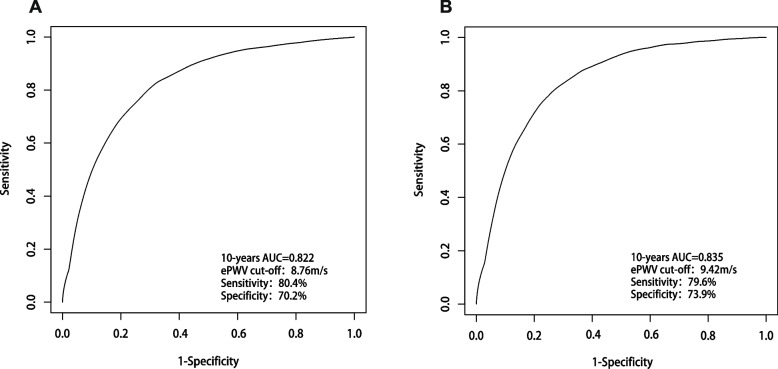
**A** Time-dependent ROC curves for ePWV prediction of 10-year all-cause mortality. **B** Time-dependent ROC curves for ePWV prediction of 10-year CVD mortality

The predictive value of ePWV for 10-year ACM and CVM was assessed based on time-dependent ROC curves. As shown in Fig. 5A, ePWV had a powerful predictive value for 10-year ACM in the general population (AUC = 0.822). The cut-off value for ePWV was 8.76 m/s, and the sensitivity and specificity were 80.4% and 70.2%, respectively. As shown in Fig. 5B, ePWV maintained a robust predictive value for 10-year CVM(AUC = 0.835) with a cut-off of 9.42 m/s. Sensitivity and specificity were 79.6% and 73.9%, respectively. Blood pressure and age are key parameters in the calculation of ePWV, the value of these parameters in predicting ACM and CVM risk was compared with ePWV. The predictive validity of age and age squared was slightly improved compared with ePWV, while the predictive value of ePWV was significantly superior to that of DBP, SBP, and MBP. For a detailed description, refer to Figure S4. However, the predictive value of the model remained essentially unchanged whether age and its square were combined with blood pressure or with MBP (see Figure S5 for details).

### Comparison of ePWV with FRS and PCE models

**Fig. 6:**
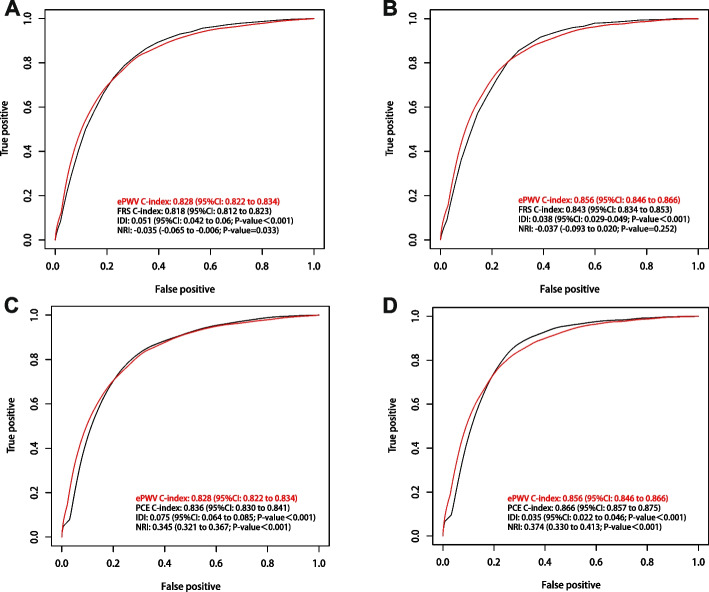
**A** Comparison of ePWV and FRS models for the risk of 10-year all-cause mortality in general population **B** Comparison of ePWV and FRS models for the risk of 10-year CVD mortality in general population. **C** Comparison of ePWV and PCE models for the risk of 10-year all-cause mortality in general population **D** Comparison of ePWV and PCE models for the risk of 10-year CVD mortality in general population. FRS, Framingham Risk Score. PCE, Pooled Cohort Equations

In the general population, the ePWV model improved the prediction of 10-year ACM by 5.1% relative to the FRS model (IDI 0.051; 95% CI 0.042–0.06; *P* < 0.001; Fig. 6A). However, no further improvement was found in the continuous NRI (NRI -0.035; *P* = 0.033). In the general population, ePWV predicted the 10-year ACM beyond the FRS model, with a median improvement in risk score of 0.4% (95% CI 0–0.008; *P* = 0.003). Meanwhile, ePWV continued to outperform the FRS in predicting 10-year CVM, with a 3.8% improvement in predictive value compared with the FRS model (IDI 0.038; 95% CI 0.029–0.049; *P* < 0.001; Fig. 6B). However, the continuous NRI did not improve further (NRI -0.037; *P* = 0.252). Finally, to predict 10-year CVM in the overall population, ePWV was shown to be superior to the FRS, with an improvement of 0.3% in the median risk score (95% CI 0–0.011; *P* = 0.047). The predictive value of ePWV for 10-year ACM and CVM was lower than that of the FRS model after participants were restricted to those aged 30–74 years with no history of CVD (ACM: IDI: -0.014, NRI: -0.179, all *p*-values < 0.001; CVM: IDI: -0.007, NRI: -0.255, all *p*-values < 0.05; Figures S6A and B).

In the general population, the ePWV model had an improvement of 5.1% in predicting 10-year ACM over the PCE model (IDI 0.075; 95% CI 0.064–0.085; *P* < 0.001;  Fig. 6C) and an improvement of 34.5% in event classification (NRI 0.345; *P* < 0.001). The prediction of 10-year ACM by ePWV exceeded the PCE model, with an improvement of 6.3% in the median risk score (95% CI 0.055–0.072; *P* < 0.001). The ePWV model outperformed the PCE model in predicting 10-year CVM, with an improvement of 3.5% in predictive value (IDI 0.035; 95% CI 0.022–0.046; *P* < 0.001; Fig. 6D) and an improvement of 37.4% in event classification (NRI 0.374; *P* < 0.001). The median risk score for 10-year CVM predicted by ePWV was improved by 2.4% (95% CI 0.019–0.030; *P* < 0.001) compared with the PCE model. However, after restricting participants to individuals aged between 30 and 79 years with no history of cardiovascular disease, the predictive value of ePWV for 10-year ACM and CVM did not significantly outperform that of the PCE model (ACM IDI: 0.006, *P* = 0.173; CVM IDI: -0.004, *P* = 0.439; Figures S6C and D). However, event classification was improved by 14.7% (NRI: 0.147, *P* < 0.001) and 13.9% (NRI: 0.139, *P* = 0.007), respectively.

## Discussion

To the best of our knowledge, this study represents the most extensive sample size for evaluating the relationship between estimated pulse wave velocity and the risk of ACM and CVM in the general population. Our findings demonstrated that ePWV served as an independent risk factor for both ACM and CVM in the general population, and was independent of traditional risk factors. The association between ePWV and the risk of ACM and CVM exhibited a non-linear pattern. In the general population aged between 20 to 85 years, ePWV demonstrated significant predictive value for the 10-year risk of ACM and CVM, irrespective of the presence or absence of cardiovascular disease. This predictive power outperformed both the FRS and the PCE models. The predictive value of ePWV for ACM and CVM appears to be more dependent on age.

Advanced age is a vital factor in natural human aging, and arterial stiffness, which develops with age, and directly affects blood and pulse pressure. Although age and blood pressure are strong predictors of mortality risk score outcomes, current risk stratification models may not be able to fully capture these complex interactions as their initial goal is to create relatively simple models [14]. ePWV is an estimate of cfPWV that has been published by the Arterial Stiffness Reference Value Collaboration [15]. It is designed to describe the relationship between cfPWV, age, and mean blood pressure in different prior cardiovascular risk groups. Considering the complex interactions and nonlinearities between age and blood pressure, ePWV provides a more comprehensive understanding of arterial stiffness and its potential impact on cardiovascular risk. This estimation allows researchers and clinicians to better assess arterial stiffness in various populations and identify individuals who may be at a higher risk of cardiovascular disease. Furthermore, the study revealed that ePWV was an independent risk factor that outperformed traditional models such as FRS and PEC in predicting ACM and CVM. Age is the most significant predictor of mortality, representing the natural progression from ageing to death, a process that cannot be interfered with. Although ePWV was slightly less valuable than age in these predictions, it was significantly superior to several types of BP indicators, including DBP, SBP and MBP. These findings underscore the potential of ePWV as an effective tool for assessing mortality. It not only helps identify high-risk individuals but also serves as a means of monitoring and modulating the effectiveness of treatment. Moreover, ePWV considers the complex and nonlinear effects of traditional risk factors on the vascular system, with various risk factors interacting with each other. It could be observed that MBP had a U-shaped relationship with the risk of ACM. As shown in Table S5, the risk of ACM was gradually increased when MBP was ≥ 82 mmHg, with a 2% increase in the risk of ACM for every 5-mmHg increase. However, in Table S4, for every 5 mmHg decrease in MBP at a constant age, ePWV was decreased by 0.15–0.2 m/s, suggesting a 7.5–10% reduction in the risk of ACM. This further supports the idea that ePWV can capture more risk. In addition, they suggest an additional benefit of lower blood pressure in lowering the risk of mortality. Our findings reinforce the critical role of ePWV in assessing mortality risk and suggest the possibility of personalized medicine. ePWV's strong correlation with age and superiority over traditional blood pressure indicators make it indispensable in developing personalized treatment plans. For instance, clinicians can use ePWV values as a reference point for more effective management, considering a patient's age and blood pressure. This approach can provide more rational treatment strategies for those with higher ePWV readings. Furthermore, understanding the relationship between ePWV, age, blood pressure, and mortality risk emphasizes the need for a holistic approach to managing cardiovascular health. This approach should consider not only traditional risk factors but also the complex interactions among them.

Although several previous studies have revealed the association between ePWV and ACM and CVM, the applicability of this association needs to be assessed in different populations. Liu et al*.* followed 13,116 general individuals for a median of 7 years, finding a 132% increase in the risk of ACM with every 1.9 m/s increase in ePWV levels [28]. In a multicenter study of 107,599 healthy individuals, Vishram-Nielsen et al*.* observed a 15% increase in the risk of ACM with every 1 m/s increase in ePWV levels, but showed no significant association with CVM [29]. Hefferman et al*.* analyzed the NHANES cohort from 1999 to 2006 and discovered that in the general population without CVD, each 1 m/s increase in ePWV was associated with a 52% and 47% increase in the risk of ACM and CVM, respectively [12]. These results reinforce the "generality" of the positive association between ePWV and ACM and the “specificity” of the risk of mortality across cohorts. This conforms to the findings by Vishram-Nielsen et al*.* that the prognostic information on ePWV varies considerably between European countries [29]. In this study, totally 32,930 general population individuals were analyzed, including those with CVD. It was found that each 1 m/s increase in ePWV was associated with a 71% and 74% increase in the risk of all-cause and CVD death, respectively. More importantly, this association remained powerful after adjusting for traditional risk models.

Markers of arterial stiffness are strongly associated with genetic markers of biological aging and life expectancy (e.g., telomere length), suggesting a common genetic susceptibility to arterial function and mortality [30]. CfPWV is the gold standard for measuring aortic stiffness, while its clinical popularity is limited by the complexity of the procedure. More recently, the study performed by Alansare et al*.* showed a strong correlation between ePWV and CfPWV (*r* = 0.7) [31]. The study by Heffernan et al*.* showed that ePWV was associated with established indicators of vascular aging such as carotid artery thickness, carotid stiffness, and increased dilatation index [32]. Furthermore, our study shows that ePWV has an excellent predictive value for 10-year ACM and CVM in the general population, with cut-off values of 8.76 m/s and 9.42 m/s, respectively, which adds to the previous studies. Greve et al*.* demonstrated that ePWV predicted major cardiovascular events independently of Systematic Coronary Risk Evaluation, Framingham risk score (FRS), and cfPWV [14]. In the SPRINT trial, ePWV predicted outcomes independently of the FRS, suggesting an incremental effect of aortic stiffness markers on cardiovascular risk [11]. In this study, we observed a nonlinear pattern in the association between ePWV and the risk of ACM and CVM, characterized by inflection points at 8.0 m/s and 7.2 m/s. The risk initially increased sharply before gradually leveling off. These results suggest that ePWV may be a useful tool in assessing vascular aging and risk. In addition, ePWV is relatively easy to obtain and can be used in the primary assessment of arterial stiffness for mortality risk stratification in cases where cfPWV cannot be measured.

MBP is a vital measure of the overall circulatory pressure load and should be maintained at least 60 mmHg to meet tissue perfusion pressure to reduce mortality caused by hypoperfusion and organ failure [33, 34]. Considering that MBP is a controlled parameter in calculating ePWV, the relationship between MBP and mortality risk was complementarily analyzed. The results showed that relative to the non-linear positive relationship between ePWV and ACM and CVM, there was a U-shaped relationship between MBP and the risk of ACM and CVM. These results reinforce that ePWV can capture mortality risk from the complex interaction between age and blood pressure better than traditional risk factors [35]. The evidence for the association between MBP and ACM risk is controversial due to regional, age, and differences in the underlying disease in the study population [36–38]. This study was conducted in the general population aged 20–85 years, exhibiting the general association between MBP and mortality in the overall population. Current guidelines for diagnosing and managing hypertension have not considered MBP as either a management target or as an indicator for calculating risk. SBP is the primary parameter representing the risk of CVD due to high blood pressure [39, 40], and more evidence is needed to confirm the clinical perspectives of MBP.

The FRS and PCE models were not originally designed to assess mortality risk. In this study, the ePWV model was compared with the FRS and PCE models in predicting 10-year ACM and CVM in the general population using two cohort populations. In the overall population, the ePWV model outperformed the FRS and PCE models, implying a broader applicability of ePWV in assessing mortality. However, the FRS outperformed the ePWV model in predicting mortality in the population aged 30 to 74 years. Among those aged 40 to 79 years, ePWV continued to outperform the PCE model in its predictive value of mortality. Although these findings further support the idea that age and blood pressure are strong predictors of mortality risk score outcomes, it is unclear how ePWV captures these interactions.

## Perspectives

The interplay between various risk factors is often neglected compared with focusing on a single traditional risk factor. In contrast to traditional risk factors, ePWV may more effectively capture additional mortality risks independent of age and blood pressure level. Given the practical concerns of complexity, cost-effectiveness, and patient tolerance associated with cfPWV measurement, ePWV may serve as an accessible and efficient alternative for monitoring arterial stiffness status. Therefore, ePWV can be employed as a “preliminary validation” and an “early warning” for individuals with higher values. Measuring cfPWV is nearly impossible in areas with limited and inadequate medical resources, while ePWV provides better clinical applicability in such regions. Furthermore, our findings emphasize the additional advantages of moderate blood pressure reduction. Due to population-specific factors, there is no uniform quantitative method for distinguishing normal and abnormal ePWV levels, which should be established in future studies.

## Limitations

Owing to the observational nature of this study, it is impossible to establish a causal relationship between ePWV and the risk of mortality. Despite adjusting for maximum covariates, the influence of residual confounders cannot be entirely ruled out. The cohort examined in this study comprised a general population from the United States, limiting the generalizability of our findings to other specific populations. The medical histories of participants were primarily self-reported, introducing the potential for subjective biases. Finally, a small portion of the baseline characteristics had missing variables (missing rate < 10%). While the results were generally consistent before and after multiple imputations, the presence of missing variables might still have an impact on our outcomes.

## Conclusion

In the U.S. adults, ePWV is an independent risk factor for ACM and CVM and is independent of traditional risk factors. In the general population aged 20 to 85 years, ePWV has a robust predictive value for the risk of all-cause and CVM risk, superior to FRS and PCE models. The predictive power of ePWV likely originates from the traditional risk factors incorporated into its calculation, rather than from an indirect association with measured pulse wave velocity.

### Supplementary Information

**Supplementary Material 1.**

## Data Availability

Data are publicly available at https://www.cdc.gov/nchs/nhanes/index.htm.
