# Supplementary material for: Superior predictive value of estimated pulse wave velocity for all-cause and cardiovascular disease mortality risk in U.S. general adults
Source: BMC Public Health. 2024 Feb 24;24:600. doi: 10.1186/s12889-024-18071-2 (PMC10893621; doi:10.1186/s12889-024-18071-2)

Supplement

Table S1. Survey-weighted cox proportional hazards results examining the association of ePWV with all-cause and CVD mortality in the general population after multiple imputation of five data sets.

Table S2. In the survey-weighted multivariate adjusted model 6, blood pressure and age were added as additional adjustments.

Table S3. Threshold-effect analysis on ePW and all-cause and CVD mortality.

Table S4. Threshold-effect analysis on mean blood pressure and all-cause and cause-specific mortality.

Table S5. ePWV values at different ages and blood pressure levels (105-75mmHg).

Figure S1. Study flow.

Figure S2: Kaplan-Meier survival curves, by ePWV quartile level, for all-cause mortality. Follow-up was initiated one years after enrollment, and landmark analyses were performed within one year of the start of follow-up.

Figure S3. Dose-response relationship between MBP with the risk of all-cause and CVD mortality. MBP, mean blood pressure

Figure S4. The ePWV model was compared with the age, age-squared, DBP, SBP, and MBP models in the cohort to determine the predictive value of the 10-year risk of all-cause and CVD mortality.

Figure S5. The ePWV model and the age combined with its squared and DBP/SBP, or MBP models were compared in the cohort to determine the predictive value of the 10-year risk of all-cause and CVD mortality.

Figure S6. Comparison of 10-year all-cause mortality risk between ePWV and FRS and PEC models.

Table S1. Survey-weighted cox proportional hazards results examining the association of ePWV with all-cause and CVD mortality in the general population after multiple imputation of five data sets.

Outcomes	Multiple imputation of five data sets					Pooled results*	
**ePWV, 1 m/s increase**	**1**	**2**	**3**	**4**	**5**	**HR (95%CI)**	**P value**
**All-cause Mortality**	1.50 (1.44-1.53)	1.48 (1.44-1.52)	1.50 (1.46-1.55)	1.48 (1.44-1.53)	1.48 (1.44-1.52)	**1.48 (1.44-1.53)**	**＜0.001**
**CVD Mortality**	1.50 (1.43-1.57)	1.47 (1.40-1.54)	1.51 (1.44-1.58)	1.48 (1.41-1.55)	1.47 (1.40-1.54)	**1.48 (1.40-1.56)**	**＜0.001**

ICD-10, International Classification of Diseases version 10.

HR indicates the change in corresponding risk of mortality for each 1m/s increase in ePWV.

*** represents the pooled results of the multiple imputations (5 data sets**)

The HRs have been fully adjusted for heart rate, pulse pressure, race, gender, poverty income ratio, body mass index, waist, estimated glomerular filtration rate, total cholesterol, high-density lipoprotein cholesterol, cardiovascular diseases, chronic kidney disease, diabetes mellitus, chronic bronchitis, hypertension, Arthritis, antihypertensives, glucose-lowering drugs, smoking, and drinking.

Table S2. In the survey-weighted multivariate adjusted model 6, blood pressure and age were added as additional adjustments.

	Death	Adjusted Model 6[1]	Adjusted Model 6[2]
**ePWV, 1 m/s increase**		**HR (95%CI)**	**HR (95%CI)**
All-cause Mortality	5,138	1.71 (1.66-1.77)	1.30 (1.24-1.35)
CVD Mortality	1,386	1.74 (1.65-1.84)	1.27 (1.08-1.50)

[1]Adjust for all variables in model 6 except for blood pressure difference. Systolic and diastolic blood pressures replaced the pulse pressure.

[2] Adjust for all variables in model 6 except for blood pressure difference. Systolic and diastolic blood pressures replaced the blood pressure difference. Age was added to the model for additional adjustment.

Table S3. Threshold-effect analysis on ePW and all-cause and CVD mortality.

Inflection-point of ePWV (m/s)	HR	95% CI	p-value	p for nonlinear
**All-cause Mortality**				＜0.001
＜8.76	1.89	1.77-2.02	＜0.001	
≥8.76	1.38	1.34-1.41	＜0.001	
**CVD Mortality**				＜0.001
＜7.57	3.07	2.22-4.24	＜0.001	
≥7.57	1.40	1.34-1.46	＜0.001	

HR has been fully adjusted for the following variables: heart rate, pulse pressure, gender, race, poverty income ratio, body mass index, waist, estimated glomerular filtration rate, total cholesterol, high-density lipoprotein cholesterol, cardiovascular diseases, chronic kidney disease, diabetes mellitus, chronic bronchitis, hypertension, Arthritis, antihypertensives, glucose-lowering drugs, smoking, and drinking.

Table S4. Threshold-effect analysis on mean blood pressure and all-cause and cause-specific mortality.

Mean blood pressure (5mmHg)	HR	95% CI	p-value	p for nonlinear
**All-cause Mortality**				0.032
＜82	0.94	0.89-0.99	0.026	
≥82	1.02	1.01-1.04	0.004	
**CVD Mortality**				0.042
＜100	0.97	0.93-1.01	0.112	
≥100	1.05	1.01-1.10	0.018	

HR has been fully adjusted for the following variables: heart rate, gender, race, poverty income ratio, body mass index, waist, estimated glomerular filtration rate, total cholesterol, high-density lipoprotein cholesterol, cardiovascular diseases, chronic kidney disease, diabetes mellitus, chronic bronchitis, hypertension, Arthritis, antihypertensives, glucose-lowering drugs, smoking, and drinking.

Table S5. ePWV values at different ages and blood pressure levels (105-75mmHg).							
MBP	105mmHg	100 mmHg	95 mmHg	90 mmHg	85 mmHg	80 mmHg	75 mmHg
AGE	**ePWV (m/s)**						
20	***7.286***	7.113	6.939	6.765	6.592	6.418	6.245
21	7.292	7.108	6.924	6.740	6.555	6.371	6.187
22	7.301	**7.107**	6.912	6.718	6.524	6.329	6.135
23	7.314	7.110	6.905	6.701	6.497	6.292	6.088
24	7.330	7.116	**6.902**	6.688	6.474	6.260	6.046
25	7.350	7.127	6.903	6.680	6.456	6.233	6.009
26	7.374	7.141	6.909	**6.676**	6.443	6.211	5.978
27	7.401	7.160	6.918	6.676	6.435	6.193	5.952
28	7.432	7.182	6.932	6.681	**6.431**	6.181	5.930
29	7.467	7.208	6.949	6.691	6.432	6.173	5.915
30	7.505	7.238	6.971	6.704	6.438	**6.171**	5.904
31	7.547	7.272	6.997	6.722	6.448	6.173	5.898
32	7.592	7.310	7.027	6.745	6.463	6.180	**5.898**
33	7.641	7.351	7.062	6.772	6.482	6.192	5.903
34	7.694	7.397	7.100	6.803	6.506	6.210	5.913
35	7.750	7.446	7.143	6.839	6.535	6.232	5.928
36	7.810	7.500	7.189	6.879	6.569	6.259	5.948
37	7.873	7.557	7.240	6.924	6.607	6.290	5.974
38	7.940	7.618	7.295	6.973	6.650	6.327	6.005
39	8.011	7.683	7.354	7.026	6.697	6.369	6.041
40	8.085	7.751	7.417	7.084	6.750	6.416	6.082
41	8.163	7.824	7.485	7.146	6.807	6.467	6.128
42	8.245	7.901	7.556	7.212	6.868	6.524	6.180
43	8.330	7.981	7.632	7.283	6.934	6.585	6.236
44	8.419	8.065	7.712	7.358	7.005	6.652	6.298
45	8.511	8.153	7.796	7.438	7.081	6.723	6.365
46	8.607	8.246	7.884	7.522	7.161	6.799	6.438
47	8.707	8.341	7.976	7.611	7.246	6.880	6.515
48	8.810	8.441	8.073	7.704	7.335	6.966	6.598
49	8.917	8.545	8.173	7.801	7.429	7.057	6.686
50	9.027	8.653	8.278	7.903	7.528	7.153	6.779
51	9.141	8.764	8.387	8.009	7.632	7.254	6.877
52	9.259	8.879	8.499	8.120	7.740	7.360	6.980
53	9.380	8.998	8.617	8.235	7.853	7.471	7.089
54	9.505	9.122	8.738	8.354	7.970	7.586	7.203

55	9.634	9.248	8.863	8.478	8.092	7.707	7.322
56	9.766	9.379	8.993	8.606	8.219	7.832	7.446
57	9.902	9.514	9.126	8.738	8.351	7.963	7.575
58	10.041	9.653	9.264	8.875	8.487	8.098	7.710
59	10.184	9.795	9.406	9.017	8.628	8.239	7.849
60	10.331	9.941	9.552	9.163	8.773	8.384	7.994
61	10.481	10.092	9.702	9.313	8.923	8.534	8.144
62	10.635	10.246	9.857	9.467	9.078	8.689	8.300
63	10.792	10.404	10.015	9.626	9.238	8.849	8.460
64	10.953	10.566	10.178	9.790	9.402	9.014	8.626
65	11.118	10.731	10.344	9.957	9.571	9.184	8.797
66	11.287	10.901	10.515	10.130	9.744	9.358	8.973
67	11.458	11.074	10.690	10.306	9.922	9.538	9.154
68	11.634	11.252	10.869	10.487	10.105	9.723	9.340
69	11.813	11.433	11.053	10.673	10.292	9.912	9.532
70	11.996	11.618	11.240	10.862	10.485	10.107	9.729
71	12.182	11.807	11.432	11.057	10.681	10.306	9.931
72	12.372	12.000	11.628	11.255	10.883	10.510	10.138
73	12.566	12.197	11.827	11.458	11.089	10.720	10.350
74	12.763	12.397	12.031	11.666	11.300	10.934	10.568
75	12.964	12.602	12.240	11.877	11.515	11.153	10.791
76	13.169	12.810	12.452	12.094	11.735	11.377	11.019
77	13.377	13.023	12.668	12.314	11.960	11.606	11.252
78	13.588	13.239	12.889	12.539	12.189	11.840	11.490
79	13.804	13.459	13.114	12.769	12.424	12.079	11.734
80	14.023	13.683	13.343	13.002	12.662	12.322	11.982
81	14.245	13.910	13.576	13.241	12.906	12.571	12.236
82	14.471	14.142	13.813	13.483	13.154	12.825	12.495
83	14.701	14.378	14.054	13.730	13.407	13.083	12.759
84	14.935	14.617	14.299	13.982	13.664	13.346	13.029
85	15.172	14.860	14.549	14.238	13.926	13.615	13.303

Figure S1. Study flow.

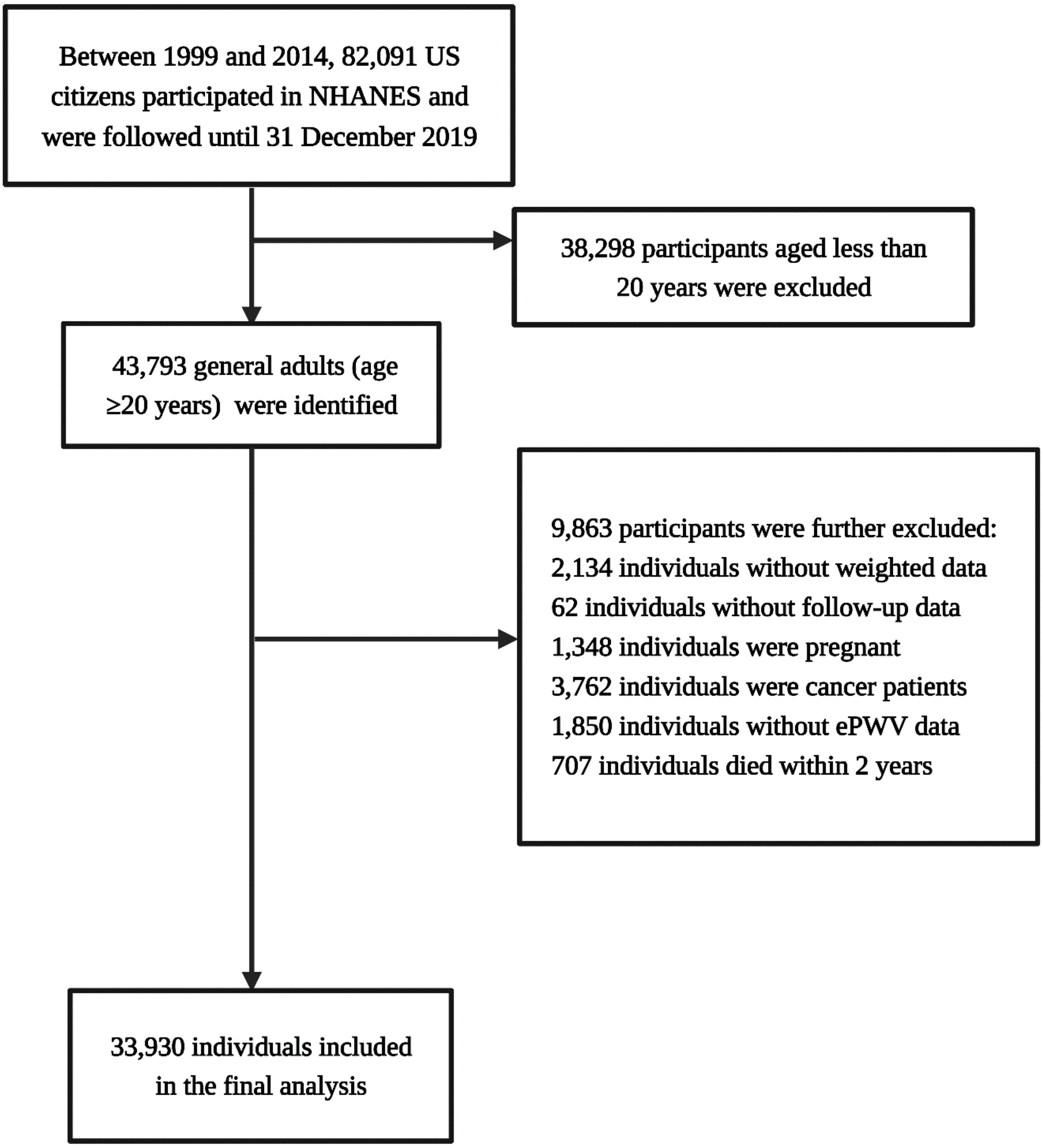

Figure S2. Kaplan-Meier survival curves, by ePWV quartile level, for all-cause mortality. Follow-up was initiated one years after enrollment, and landmark analyses were performed within one year of the start of follow-up.

In the multivariate model the HRs have been fully adjusted for heart rate, pulse pressure, race, gender, poverty income ratio, body mass index, waist, estimated glomerular filtration rate, total cholesterol, high-density lipoprotein cholesterol, cardiovascular diseases, chronic kidney disease, diabetes mellitus, chronic bronchitis, hypertension, Arthritis, antihypertensives, glucose-lowering drugs, smoking, and drinking.

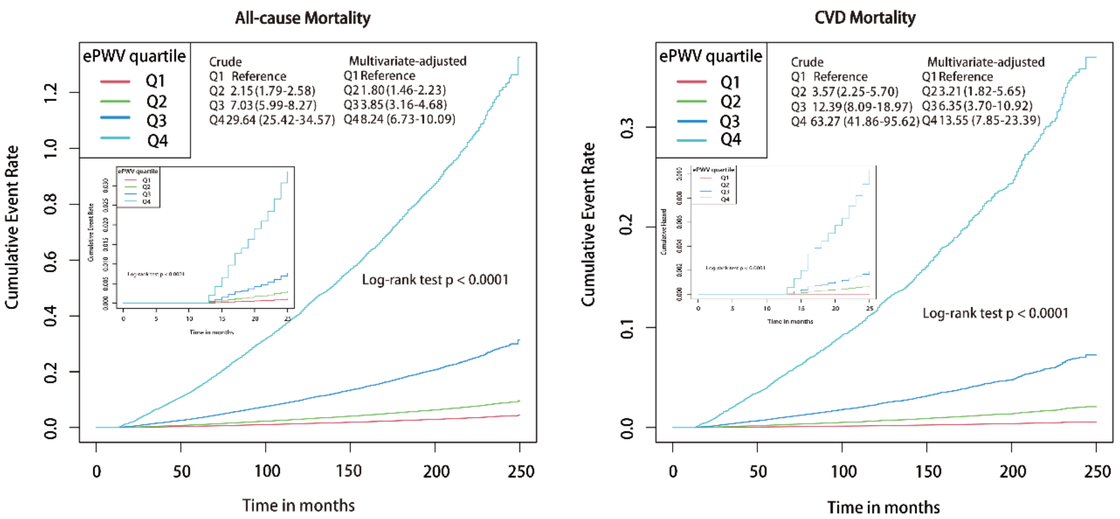

Figure S3. Dose-response relationship between MBP with the risk of all-cause and CVD mortality. MBP, mean blood pressure.

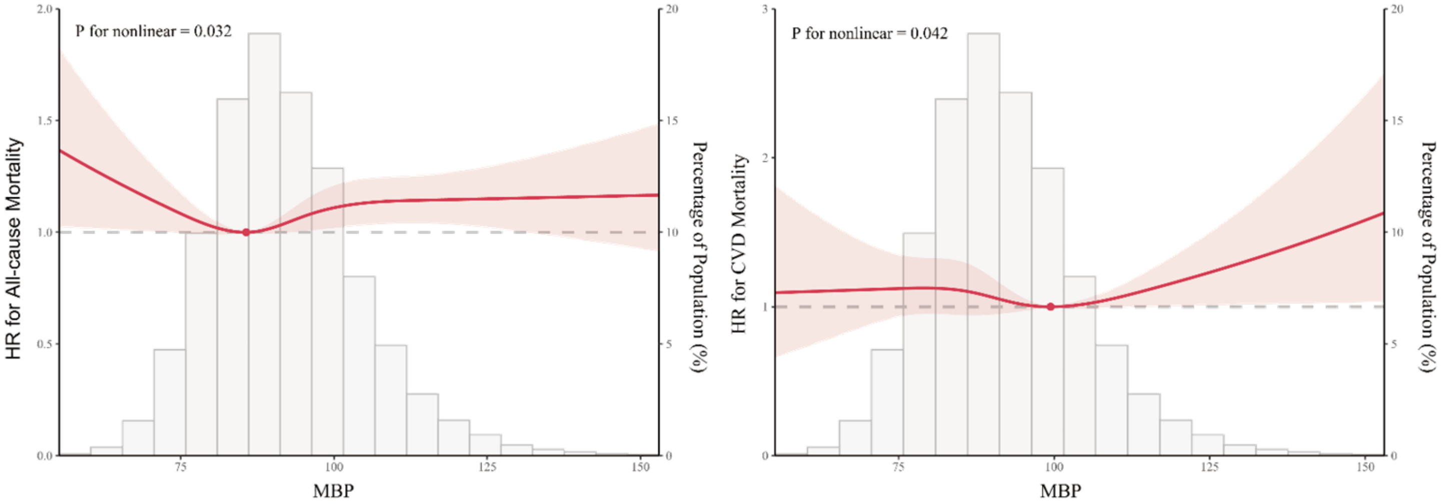

Figure S4. The ePWV model was compared with the age, age-squared, DBP, SBP, and MBP models in the cohort to determine the predictive value of the 10-year risk of all-cause and CVD mortality.

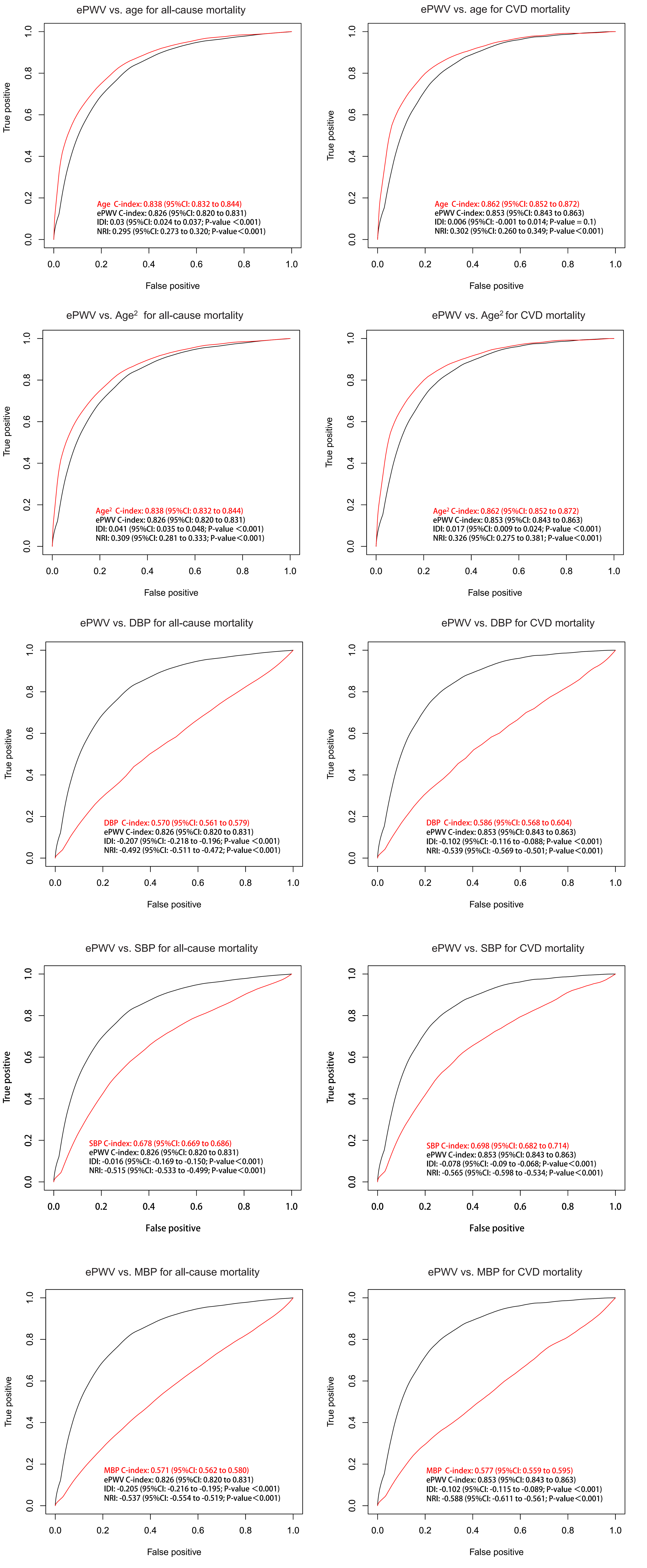

Figure S5. The ePWV model and the age combined with its squared and DBP/SBP, or MBP models were compared in the cohort to determine the predictive value of the 10-year risk of all-cause and CVD mortality.

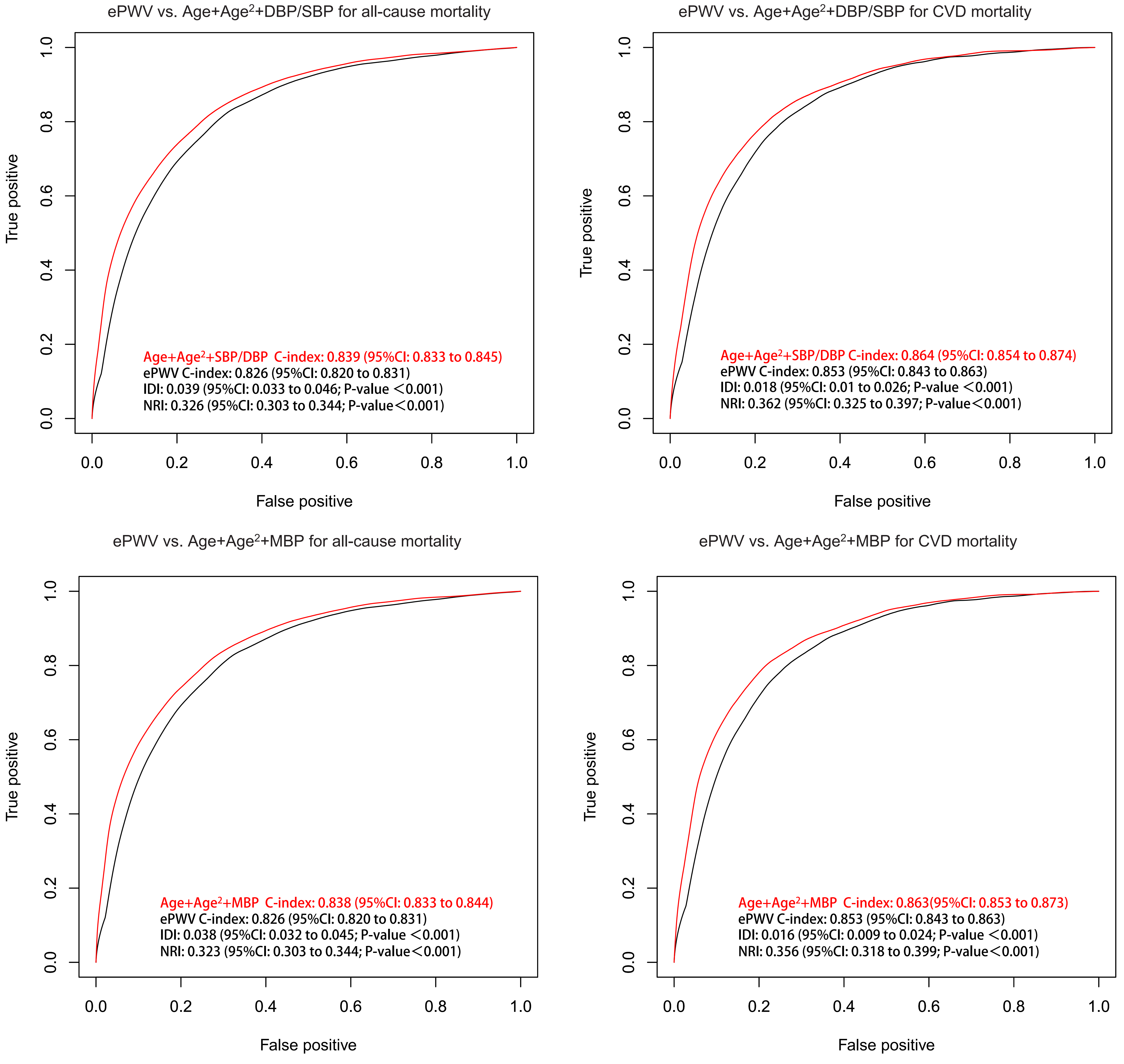

Figure S6.

A. Comparison of 10-year all-cause mortality risk between ePWV and FRS models in the cohort of 30-74 years.

B. Comparison of 10-year CVD mortality risk between ePWV and FRS models in the cohort of 30-74 years.

C. Comparison of 10-year all-cause mortality risk between the ePWV and PCE models in the cohort of 40-79 years.

D. Comparison of 10-year CVD mortality risk between the ePWV and PCE models in the cohort of 40-79 years.

FRS, Framingham Risk Score. PCE, Pooled Cohort Equation.

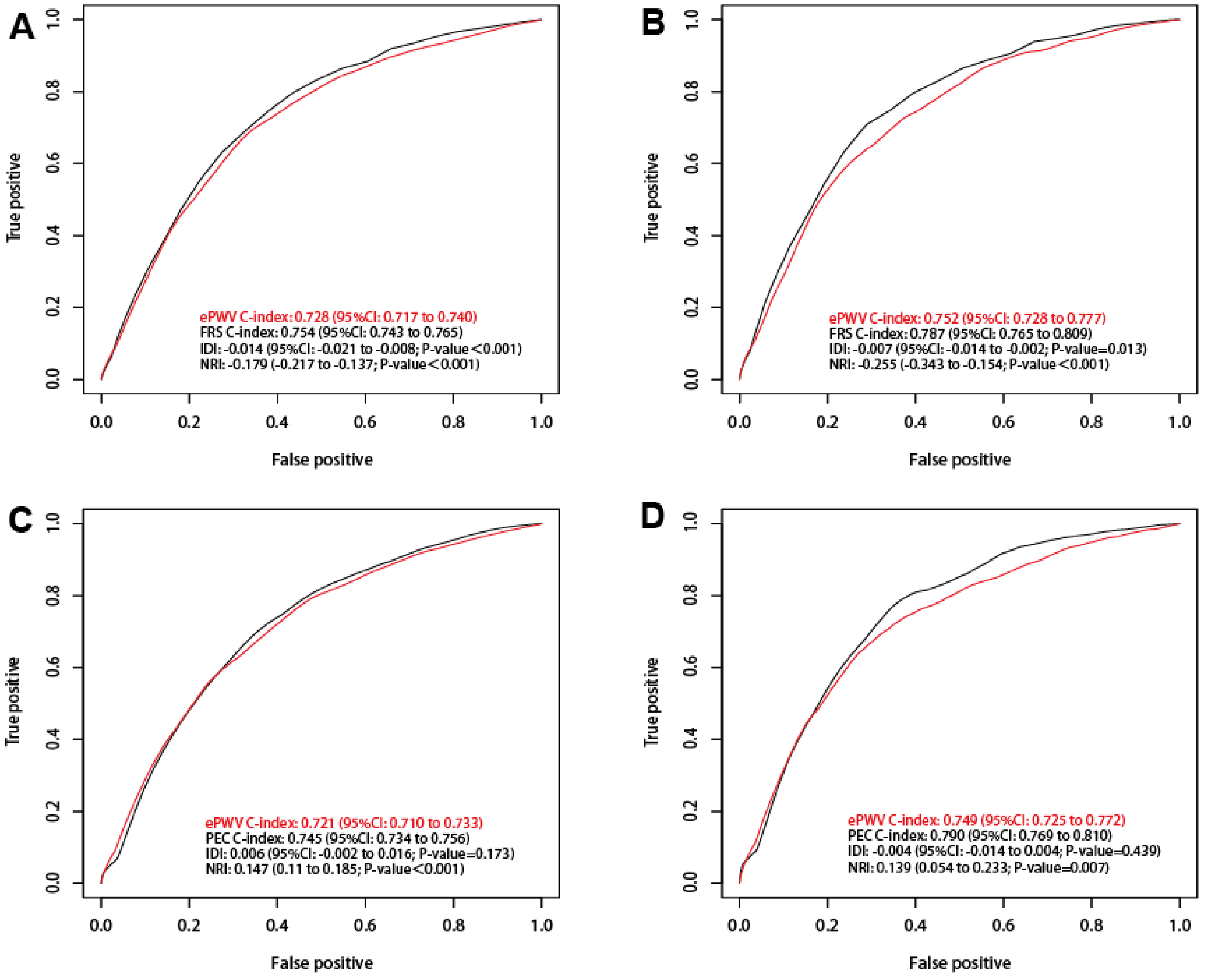

Supplement: Supplementary file 1 — Supplementary Material 1. [file 12889_2024_18071_MOESM1_ESM.pdf]
